# Human leukocyte antigen class I (A, B and C) allele and haplotype variation in a South African Mixed ancestry population

**DOI:** 10.1016/j.humimm.2017.04.006

**Published:** 2017

**Authors:** Shayne Loubser, Maria Paximadis, Caroline T. Tiemessen

**Affiliations:** Centre for HIV-1 and STIs, National Institute for Communicable Diseases, National Health Laboratory Services and Faculty of Health Sciences, University of the Witwatersrand, Johannesburg, South Africa

**Keywords:** HLA alleles, HLA haplotypes, South African population, Mixed ancestry, Coloured

## Abstract

South Africa has a large (∼53 million), ethnically diverse population (black African, Caucasian, Indian/Asian and Mixed ancestry) and a high disease burden (particularly HIV-1 and *Mycobacterium tuberculosis*). The Mixed ancestry population constitutes ∼9% of the total population and was established ∼365 years ago in the Western Cape region through interracial mixing of black Africans, Europeans and Asians. Admixed populations present unique opportunities to identify genetic factors involved in disease susceptibility. Since *HLA* genes are important mediators of host immunity, we investigated *HLA-A*, -*B* and -*C* allele and haplotype diversity in 50 healthy, unrelated individuals recruited from the Mixed ancestry population.

South Africa, located at the most southerly tip of Africa, has a unique demographic history and ethnic diversity, due to extensive migration of people from the northern parts of Africa and also via oceanic trade-routes between the West and the East. The southern African region was first populated by the Khoi-San, before Bantu-speaking tribes migrated into the area ∼2000 years ago. European colonisation of the Western Cape region began in the mid-1600′s and included Dutch, German, English and French settlers as well as slave labour imported from Indonesia, India, Madagascar and East Africa. The South African Mixed ancestry (SAM) or “Coloured” population was established through interracial mixing of Europeans, slaves and the indigenous Khoi-San and Xhosa tribes. In 2013, the South African population was recorded at 52,982,000 individuals, divided into four major population groups (black African, Caucasian, Indian/Asian and Mixed ancestry) (statssa.gov.za/publications/P0302/P03022013.pdf). The predominantly Afrikaans (afr)-speaking SAM population (∼80%), but also English (eng)-speaking, mostly reside in the Western Cape Province and constitute ∼9% of the total population [1].

Several genetic studies interrogating autosomes agree with historical records of early Western Cape demographics that contributed to the genetic admixture of the SAM population [1], [2], [3], [4], [5]. Mitochondrial DNA and Y-chromosome analyses also confirm at least five genetically distinct lineages involving Khoi-San, Bantu, European, Indian and Southeast Asian sources, and further reveal a gender biased admixture showing enrichment of Khoi-San maternally inherited mitochondrial DNA, while Y-chromosomes originated primarily from European and African males [3], [6].

HIV-1 and *Mycobacterium tuberculosis* (*Mtb*) infection are the most common infectious diseases and leading causes of death in South Africa. In the SAM population, HIV-1 prevalence was 3.1% in 2012, well below the national prevalence of 12.2% (hsrc.ac.za/en/research-outputs/view/6871), while in 2013, *Mtb* was the second leading cause of death (7.1%) (statssa.gov.za/publications/P03093/P030932013.pdf). Differential susceptibility to infectious diseases suggests a role for host immunogenetic factors. Indeed, specific *HLA* alleles associate with both *Mtb* [7] and HIV-1 infection [8].

We investigated *HLA-A*, -*B* and -*C* allele and haplotype variation in 50 healthy, unrelated individuals recruited from the SAM population. Buffy coats stored at −80 °C were available for 40 individuals from the Electricity Supply Commission (ESKOM) cohort [9], while an additional 10 volunteers were recruited from staff/students at the National Institute for Communicable Diseases, the University of the Witwatersrand (WITS) Medical School and the WITS Donald Gordon Medical Centre in Johannesburg. Ethnicity was self-determined by each participant and signed consent was also obtained. Ethics approval was granted by the Human Research Ethics Committee (Medical) of the WITS medical faculty.

Genomic DNA was extracted from buffy coats or fresh whole blood using the QiaAmp DNA blood mini kit (Qiagen, Hilden, Germany) and quantified using a nanodrop spectrophotometer (Thermo Scientific, Waltham, USA). The sequence-based typing (SBT) resolver kit (Conexio Genomics, Fremantle, Australia) was used to generate *HLA-A, -B* and -*C* amplicons for DNA sequencing as described by the manufacturers. PCR products were purified using Agencourt Ampure XP magnetic bead separation (Becton-Dickenson, Franklin lakes, USA). Sequence products were generated using the BigDye terminator v3.1 cycle sequencing kit (Life Technologies, Carlsbad, USA), purified using ethanol-sodium acetate precipitation and resolved on an ABI3100 PRISM Genetic Analyser (Life Technologies, Carlsbad, USA). Alleles were assigned using ASSIGN-SBT v4.7 software (Conexio Genomics, Fremantle, Australia) and v3.13.1.4 of the IPD-IMGT/HLA reference database released on July 25, 2013. No additional molecular-based methods were employed to resolve ambiguities, however, the following steps were taken to exclude unlikely allele combinations from the ambiguity list: (i) analysis using the SBT software was initially used interrogate differences in exons 1–4 for *HLA-A*/*B* and exons 1–8 for *HLA-C*, (ii) since partial *5′UTR* and introns 1,2 for *HLA-A/B* and partial *5′UTR*, introns 1,2,4–7 and partial *3′UTR* sequences were also generated, these differences could also be analysed by manual editing using the SBT software, (iii) likely allele combinations were based on “Common” and “Well-Documented” alleles listed in version 2.0.0 of the “Common and Well-Documented HLA alleles catalogue” updated in 2012 [10], (iv) in some cases, other studies investigating the same population [7] or ancestral populations such as South African black and Caucasian populations [9] were consulted and finally, (v) haplotype data available on the Allele Frequency Net Database (AFND) was also used to confirm linkage of *HLA-A, -B* and *-C* alleles [11]. Allele frequencies, haplotypes and deviations from Hardy-Weinberg equilibrium were calculated using the PyPop-Win32-0.7.0 software program available online (www.pypop.org) [12].

Overall, no deviations from Hardy-Weinberg equilibrium were detected at *HLA-A*, *-B* and *-C* loci (p = 0.1852, p = 0.4713 and p = 0.3362, respectively). The number of unique *HLA-A*, -*B* and -*C* alleles identified was n = 24, n = 43 and n = 25, respectively (Table S1). HLA-A*02:01:01:01, -B*07:02:01 and -C*06:02:01:01 were the most common alleles detected at frequencies of 0.21, 0.08 and 0.12, respectively (Table S1). HLA-A*03:01:01:01-B*07:02:01-C*07:02:01:01 was the most common haplotype detected at a frequency of 0.04, from a total of 81 unique haplotypes identified (Table S2).

The SAM population is a relatively understudied ethnic group of South Africa, and this report may contribute to a better understanding of disease susceptibility related to immune function. Data has been deposited in the AFND database under the identifier 3395.
