# Supplementary material for: Human leukocyte antigen class I (A, B and C) allele and haplotype variation in a South African Mixed ancestry population
Source: Hum Immunol. 2017 May-Jun;78(5-6):399–400. doi: 10.1016/j.humimm.2017.04.006 (PMC5451074; doi:10.1016/j.humimm.2017.04.006)
Supplement: Supplementary data [file mmc1.docx]

**Table S1: *HLA-A*, -*B* and -*C* allele frequencies determined for the South African Mixed ancestry population (n=50).**

| **A allele** | **Freq.** | **Counts** | **B allele** | **Freq.** | **Counts** | **C allele** | **Freq.** | **Counts** |
| --- | --- | --- | --- | --- | --- | --- | --- | --- |
| 01:01:01:01 | 0.12 | 12 | 07:02:01 | 0.08 | 8 | 01:02:01 | 0.02 | 2 |
| 02:01:01:01 | 0.21 | 21 | 08:01:01 | 0.02 | 2 | 02:02:02 | 0.04 | 4 |
| 02:03:01 | 0.01 | 1 | 13:01:01 | 0.01 | 1 | 02:10 | 0.02 | 2 |
| 02:11:01 | 0.02 | 2 | 14:01:01 | 0.02 | 2 | 03:02:02:01 | 0.01 | 1 |
| 03:01:01:01 | 0.06 | 6 | 14:02:01 | 0.02 | 2 | 03:03:01 | 0.01 | 1 |
| 11:01:01 | 0.06 | 6 | 15:01:01:01 | 0.03 | 3 | 03:04:01:01 | 0.07 | 7 |
| 23:01:01 | 0.04 | 4 | 15:02:01 | 0.01 | 1 | 04:01:01:01 | 0.09 | 9 |
| 24:02:01:01 | 0.09 | 9 | 15:03:01 | 0.03 | 3 | 04:03:01 | 0.01 | 1 |
| 24:07 | 0.04 | 4 | 15:10:01 | 0.02 | 2 | 05:01:01:01 | 0.02 | 2 |
| 25:01:01 | 0.02 | 2 | 15:13:01 | 0.02 | 2 | 06:02:01:01 | 0.12 | 12 |
| 26:01:01 | 0.02 | 2 | 15:16:01 | 0.01 | 1 | 07:01:01:01 | 0.07 | 7 |
| 29:02:01:01 | 0.04 | 4 | 18:01:01:01 | 0.07 | 7 | 07:02:01:01 | 0.09 | 9 |
| 30:01:01 | 0.04 | 4 | 27:05:02 | 0.03 | 3 | 07:04:01 | 0.04 | 4 |
| 30:02:01 | 0.05 | 5 | 35:01:01:01 | 0.01 | 1 | 07:06 | 0.01 | 1 |
| 30:04:01 | 0.02 | 2 | 35:02:01 | 0.01 | 1 | 08:01:01 | 0.04 | 4 |
| 31:01:02 | 0.01 | 1 | 35:03:01 | 0.03 | 3 | 08:02:01 | 0.04 | 4 |
| 32:01:01 | 0.01 | 1 | 35:05:01 | 0.02 | 2 | 12:02:02 | 0.02 | 2 |
| 33:03:01 | 0.01 | 1 | 37:01:01 | 0.01 | 1 | 12:03:01:01 | 0.06 | 6 |
| 34:01:01 | 0.01 | 1 | 38:01:01 | 0.01 | 1 | 14:02:01 | 0.01 | 1 |
| 34:02:01 | 0.03 | 3 | 38:02:01 | 0.01 | 1 | 15:02:01 | 0.03 | 3 |
| 43:01 | 0.02 | 2 | 39:06:02 | 0.01 | 1 | 15:05:02 | 0.01 | 1 |
| 66:01 | 0.01 | 1 | 39:10:01 | 0.02 | 2 | 16:01:01 | 0.04 | 4 |
| 68:01:02 | 0.02 | 2 | 40:01:02 | 0.04 | 4 | 17:01:01:02 | 0.07 | 7 |
| 68:02:01:01 | 0.04 | 4 | 40:02:01 | 0.02 | 2 | 18:01 | 0.01 | 1 |
|  |  |  | 40:06:01:01 | 0.03 | 3 | 18:02 | 0.05 | 5 |
|  |  |  | 41:01 | 0.02 | 2 |  |  |  |
|  |  |  | 42:01:01 | 0.05 | 5 |  |  |  |
|  |  |  | 44:03:01 | 0.03 | 3 |  |  |  |
|  |  |  | 44:03:02 | 0.01 | 1 |  |  |  |
|  |  |  | 44:05:01 | 0.02 | 2 |  |  |  |
|  |  |  | 44:29 | 0.01 | 1 |  |  |  |
|  |  |  | 45:01 | 0.03 | 3 |  |  |  |
|  |  |  | 47:01:01:01 | 0.01 | 1 |  |  |  |
|  |  |  | 49:01:01 | 0.03 | 3 |  |  |  |
|  |  |  | 51:01:01 | 0.04 | 4 |  |  |  |
|  |  |  | 52:01:01:01 | 0.02 | 2 |  |  |  |
|  |  |  | 53:01:01 | 0.01 | 1 |  |  |  |
|  |  |  | 57:01:01 | 0.04 | 4 |  |  |  |
|  |  |  | 57:02:01 | 0.01 | 1 |  |  |  |
|  |  |  | 58:01:01 | 0.02 | 2 |  |  |  |
|  |  |  | 58:02 | 0.05 | 5 |  |  |  |
|  |  |  | 81:01 | 0.01 | 1 |  |  |  |

**Table S2: *HLA-ABC* haplotype frequencies determined for the South African Mixed ancestry population (n=50).**

| **A allele** | **B allele** | **C allele** | **Frequency**  **(%)** | **Counts** |
| --- | --- | --- | --- | --- |
| 03:01:01:01 | 07:02:01 | 07:02:01 | 4 | 4 |
| 30:01:01 | 42:01:01 | 17:01:01:02 | 3 | 3 |
| 01:01:01:01 | 57:01:01 | 06:02:01:01 | 3 | 3 |
| 02:01:01:01 | 40:06:01:01 | 15:02:01 | 3 | 3 |
| 02:01:01:01 | 07:02:01 | 07:02:01:01 | 2 | 2 |
| 68:02:01:01 | 14:01:01 | 08:02:01 | 2 | 2 |
| 11:01:01 | 18:01:01:01 | 03:04:01:01 | 2 | 2 |
| 24:07 | 35:05:01 | 04:01:01:01 | 2 | 2 |
| 43:01 | 15:03:01 | 18:02 | 2 | 2 |
| 29:02:01:01 | 42:01:01 | 17:01:01:02 | 2 | 2 |
| 68:02:01:01 | 14:02:01 | 08:02:01 | 2 | 2 |
| 34:02:01 | 58:02 | 18:02 | 2 | 2 |
| 01:01:01:01 | 08:01:01 | 07:01:01:01 | 2 | 2 |
| 02:01:01:01 | 44:05:01 | 02:02:02 | 2 | 2 |
| 01:01:01:01 | 37:01:01 | 14:02:01 | 1 | 1 |
| 02:01:01:01 | 15:16:01 | 06:02:01:01 | 1 | 1 |
| 24:02:01:01 | 58:02 | 06:02:01:01 | 1 | 1 |
| 03:01:01:01 | 40:01:02 | 03:04:01:01 | 1 | 1 |
| 34:02:01 | 44:03:01 | 04:01:01:01 | 1 | 1 |
| 23:01:01 | 45:01 | 06:02:01:01 | 1 | 1 |
| 02:01:01:01 | 41:01 | 17:01:01:02 | 1 | 1 |
| 24:02:01:01 | 18:01:01:01 | 07:01:01:01 | 1 | 1 |
| 11:01:01 | 18:01:01:01 | 07:06 | 1 | 1 |
| 68:01:02 | 44:03:02 | 12:03:01:01 | 1 | 1 |
| 30:04:01 | 58:02 | 06:02:01:01 | 1 | 1 |
| 66:01 | 58:02 | 06:02:01:01 | 1 | 1 |
| 11:01:01 | 40:01:02 | 07:04:01 | 1 | 1 |
| 01:01:01:01 | 44:29 | 05:01:01:01 | 1 | 1 |
| 25:01:01 | 39:10:01 | 12:03:01:01 | 1 | 1 |
| 30:04:01 | 18:01:01:01 | 15:05:02 | 1 | 1 |
| 02:01:01:01 | 27:05:02 | 02:02:02 | 1 | 1 |
| 02:01:01:01 | 44:03:01 | 16:01:01 | 1 | 1 |
| 02:01:01:01 | 15:01:01:01 | 12:02:02 | 1 | 1 |
| 24:07 | 52:01:01:01 | 03:04:01:01 | 1 | 1 |
| 02:11:01 | 51:01:01 | 07:02:01:01 | 1 | 1 |
| 34:01:01 | 57:01:01 | 06:02:01:01 | 1 | 1 |
| 01:01:01:01 | 15:02:01 | 18:01 | 1 | 1 |
| 24:07 | 81:01 | 08:01:01 | 1 | 1 |
| 01:01:01:01 | 44:03:01 | 04:01:01:01 | 1 | 1 |
| 02:01:01:01 | 51:01:01 | 01:02:01 | 1 | 1 |
| 30:02:01 | 58:01:01 | 03:02:02:01 | 1 | 1 |
| 30:02:01 | 57:02:01 | 18:02 | 1 | 1 |
| 02:03:01 | 13:01:01 | 04:03:01 | 1 | 1 |
| 11:01:01 | 38:02:01 | 07:02:01:01 | 1 | 1 |
| 26:01:01 | 41:01 | 17:01:01:02 | 1 | 1 |
| 02:01:01:01 | 27:05:02 | 07:04:01 | 1 | 1 |
| 32:01:01 | 07:02:01 | 02:10 | 1 | 1 |
| 24:02:01:01 | 35:03:01 | 04:01:01:01 | 1 | 1 |
| 02:01:01:01 | 18:01:01:01 | 05:01:01:01 | 1 | 1 |
| 24:02:01:01 | 51:01:02 | 03:04:01:01 | 1 | 1 |
| 33:03:01 | 51:01:01 | 07:01:01:01 | 1 | 1 |
| 01:01:01:01 | 53:01:01 | 07:04:01 | 1 | 1 |
| 68:01:02 | 18:01:01:01 | 16:01:01 | 1 | 1 |
| 11:01:01 | 15:13:01 | 08:01:01 | 1 | 1 |
| 23:01:01 | 40:02:01 | 07:04:01 | 1 | 1 |
| 24:02:01:01 | 58:01:01 | 12:03:01:01 | 1 | 1 |
| 26:01:01 | 35:03:01 | 06:02:01:01 | 1 | 1 |
| 02:01:01:01 | 15:13:01 | 08:01:01 | 1 | 1 |
| 24:02:01:01 | 15:01:01:01 | 08:01:01 | 1 | 1 |
| 02:11:01 | 49:01:01 | 07:01:01:01 | 1 | 1 |
| 01:01:01:01 | 35:02:01 | 04:01:01:01 | 1 | 1 |
| 30:02:01 | 45:01 | 16:01:01 | 1 | 1 |
| 30:02:01 | 40:01:02 | 12:03:01:01 | 1 | 1 |
| 31:01:02 | 39:10:01 | 03:04:01:01 | 1 | 1 |
| 02:01:01:01 | 15:10:01 | 16:01:01 | 1 | 1 |
| 29:02:01:01 | 15:01:01:01 | 03:03:01 | 1 | 1 |
| 02:01:01:01 | 38:01:01 | 12:03:01:01 | 1 | 1 |
| 23:01:01 | 52:01:01:01 | 12:02:02 | 1 | 1 |
| 24:02:01:01 | 07:02:01 | 02:10 | 1 | 1 |
| 24:02:01:01 | 40:01:02 | 03:04:01:01 | 1 | 1 |
| 24:02:01:01 | 39:06:02 | 07:02:01:01 | 1 | 1 |
| 29:02:01:01 | 45:01 | 06:02:01:01 | 1 | 1 |
| 25:01:01 | 35:01:01:01 | 01:02:01 | 1 | 1 |
| 30:01:01 | 27:05:02 | 04:01:01:01 | 1 | 1 |
| 30:02:01 | 15:10:01 | 04:01:01:01 | 1 | 1 |
| 01:01:01:01 | 49:01:01 | 07:01:01:01 | 1 | 1 |
| 02:01:01:01 | 15:03:01 | 02:02:02 | 1 | 1 |
| 23:01:01 | 40:02:01 | 04:01:01:01 | 1 | 1 |
| 02:01:01:01 | 47:01:01:01 | 07:01:01:01 | 1 | 1 |
| 03:01:01:01 | 49:01:01 | 06:02:01:01 | 1 | 1 |
| 02:01:01:01 | 35:03:01 | 12:03:01:01 | 1 | 1 |
